# Transcriptomic and metabolomic changes triggered by *Macrosiphum rosivorum* in rose (*Rosa longicuspis*)

**DOI:** 10.1186/s12864-021-08198-6

**Published:** 2021-12-09

**Authors:** Penghua Gao, Hao Zhang, Huijun Yan, Ningning Zhou, Bo Yan, Yuanlan Fan, Kaixue Tang, Xianqin Qiu

**Affiliations:** 1 Flower Research Institute, Yunnan Academy of Agricultural Sciences/National Engineering Research Center for Ornamental Horticulture, Kunming, 650205 China; 2 Southwest Forestry University, Kunming, 650024 China

**Keywords:** Rose, Aphid, Glutathione metabolism, Glucosinolate metabolism

## Abstract

**Background:**

Rose is one of the most popular flowers in the wold. Its field growth and quality are negatively affected by aphids. However, the defence mechanisms used by rose plants against aphids are unclear. Therefore, to understand the defence mechanism of rose under aphid stress, transcriptome and metabolome techniques were used to investigate the regulation mechanism in *R. longicuspis* infected with *M. rosivorum.*

**Result:**

In our study, after inoculation with *M. rosivorum*, *M. rosivorum* quickly colonized *R. longicuspis*. A total of 34,202 genes and 758 metabolites were detected in all samples. Under *M. rosivorum* stress, *R. longicuspis* responded by MAPK cascades, plant hormone signal transduction pathway activation, *RlMYBs* and *RlERFs* transcription factors expression and ROS production. Interestingly, the ‘brassinosteroid biosynthesis’ pathway was significantly enriched in A3 d-vs.-A5 d. Further analysis showed that *M. rosivorum* induced the biosynthesis of secondary metabolites such as terpenoids, tannins and phenolic acids, among others. Importantly, the ‘glutathione metabolic’ and ‘glucosinolate biosynthesis’ pathways were significantly enriched, which involved in the rose against aphids.

**Conclusion:**

Our study provides candidate genes and metabolites for *Rosa* defence against aphids. This study provides a theoretical basis for further exploring the molecular regulation mechanism of rose aphid resistance and aphid resistance breeding in the future.

**Supplementary Information:**

The online version contains supplementary material available at 10.1186/s12864-021-08198-6.

## Background

Rose is one of the most important cut flowers in the world and has high ornamental and economic value [1]. Unfortunately, roses have become prey for many pests due to their high carbohydrate and sugar contents. Among various pests, aphids are the most common predator and affect the yield, quality and ornamental value of rose [2].

Aphids often gather in the immature tissues of roses and damage the immature leaves, shoots, branches and buds of roses by sucking the juice, causing leaf curling, yellowing or abnormal flowering [3]. These aphid species include *Macrosiphum rosae*, *Macrosiphum rosivorum*, *Myzus persicae*, *Myzaphis rosarum* and *Aphis gossypii.* Among them, *Macrosiphum rosivorum* is the most common and serious in rose [4, 5]. At present, insecticides such as pymetrozine and imidacloprid are mainly used to control aphids on rose crops. However, aphids have developed a tolerance to these products due to the long-term use of chemical drugs. The worse the effect, the larger the drug dosage, which not only increases the economic cost but also aggravates environmental pollution. Therefore, it is urgent to find an ecologically sustainable development method to enhance the resistance of rose to aphids.

With biotechnology developments, whole-genome sequencing of many plant species has been completed, and the results have been applied to study plant growth, environmental interactions and metabolism, among others. In recent years, transcriptomics and metabonomics have been used to explore the defence mechanisms of plant diseases and insect pests, such as rose, maize, and sesame [6–8], thus providing new insights and methods for exploring the mechanism of resistance of rose to aphids.

Aphids are phloem-feeding insects that enter plants through the epidermis and mesophyll using stylet-like mouthparts [9]. During aphid feeding, they secrete saliva containing a series of signals that stimulate the host to generate reactive oxygen species (ROS), thus leading to intracellular oxidative damage [10–13]. In previous studies, the mechanism of plant defence against aphids can be divided into two types: constructive defence and induced defence. Constitutive defence is a direct defence mechanism, which refers to the defence characteristics of plants that affect aphid feeding behaviour before aphid invasion, such as thorns, wax and trichomes. The accumulation of lignin thickens the cell wall and makes the cambium super lignified, which constitutes a mechanical barrier to insect feeding [14–16]. Induced defence is a defence characteristic of plants that is activated after an aphid attack, and plants produce volatile substances to attract the natural enemies of aphids or induce physiological and biochemical changes to defend themselves from aphids [17–19].

The germplasm resources of rose in China are rich and have high production and application value [20]. *Rosa longicuspis* is a climbing shrub of the Rosaceae family [21]. *R. longicuspis* is widely distributed in the mountainous areas of Southwest China. It has many excellent characteristics, such as the appearance of beautiful flowers, a long flowering period and strong disease resistance. It is an important germplasm resource for vines and gathering types of flowers. It is also an excellent vertical greening material that is very suitable for family planting and has good application prospects for garden flowers [22]. Moreover, our previous studies found that *R. longicuspis* is a species that is highly resistant to aphids.

The research progress on rose aphids mainly includes three aspects: first, screening and identification of rose aphid-resistant germplasm resources [5]; second, the effect of chemical drugs applied to *Rosa* plants on aphid resistance [23]; and third, the proteomics of aphid resistance in *Rosa* plants [2]. However, few reports on the changes in transcription and metabolites of rose under aphid stress. Therefore, *Macrosiphum rosivorum* was inoculated with highly resistant species of *R. longicuspis*. and the mechanism of resistance of *Rosa* to aphids was explored by transcriptional and metabolic sequencing.

## Results

### Aphid population statistics and leaf morphological changes after inoculation with *M. rosivorum* on *R. longicuspis*

**Fig. 1:**
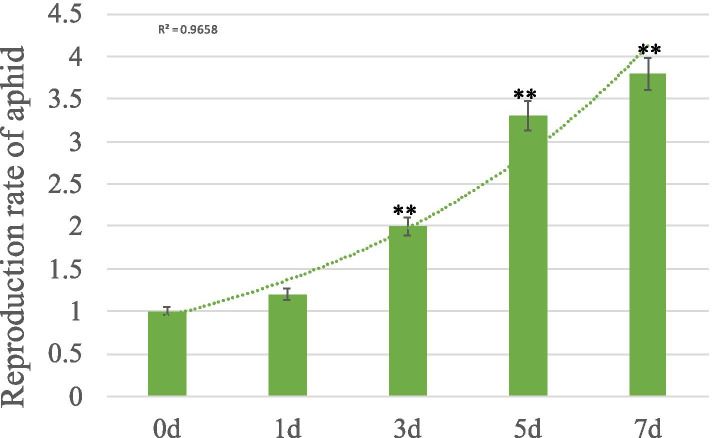
*Macrosiphum rosivorum* rate of reproduction of *Rosa longicuspis.* All statistical analyses were performed using Student’s t-test; ** indicate significant differences at the *P* < 0.01 level

To understand the reproduction of aphids, we recorded the number of aphids at 0 d, 1 d, 3 d, 5 d and 7 d. The results showed that the number of aphids increased exponentially and after inoculation for 3 d, 5 d and 7 d, the number of aphids was 2 times, 3.3 times and 3.8 times that of the first inoculation, respectively (Fig. 1). These results indicate that *M. rosivorum* has high reproducibility and ultimately harms the growth conditions of *R. longicuspis.* According to the changes in the aphid population after aphid inoculation, we selected the samples at 0 d, 3 d and 5 d as the research materials and analysed the RNA-seq, qPCR and metabonomics to explore the early mechanism of *Rosa* plants responding to aphid stress.

### Overview of the transcriptomic analysis

To further understand the molecular mechanism underlying the response of *R. longicuspis* to *M. rosivorum,* an RNA-seq analysis was performed. A total of approximately 58.22 GB clean reads were generated from nine biological samples, including six infected and three control samples. The average Q20 value of the raw reads was 95.88% and approximately 84% of the reads were mapped to the reference genome sequences obtained by Trinity splicing. In addition, more than 90.68% of the reads were mapped to the exon region of the reference genome (Table S1). Ultimately, the sequence and expression information of 34,202 genes was obtained for subsequent analysis. A principal component analysis (PCA) and Pearson correlation coefficient analysis of the samples based on the Fragments Per Kilobase per Million (FPKM) values showed that all the biological replicates exhibited similar expression patterns, indicating the high reliability of our sequencing data (Fig. S1). Taken together, the sequencing quality was sufficient for further analysis.

### Identification of DEGs in *R. longicuspis inoculated with M. rosivorum*

**Fig. 2:**
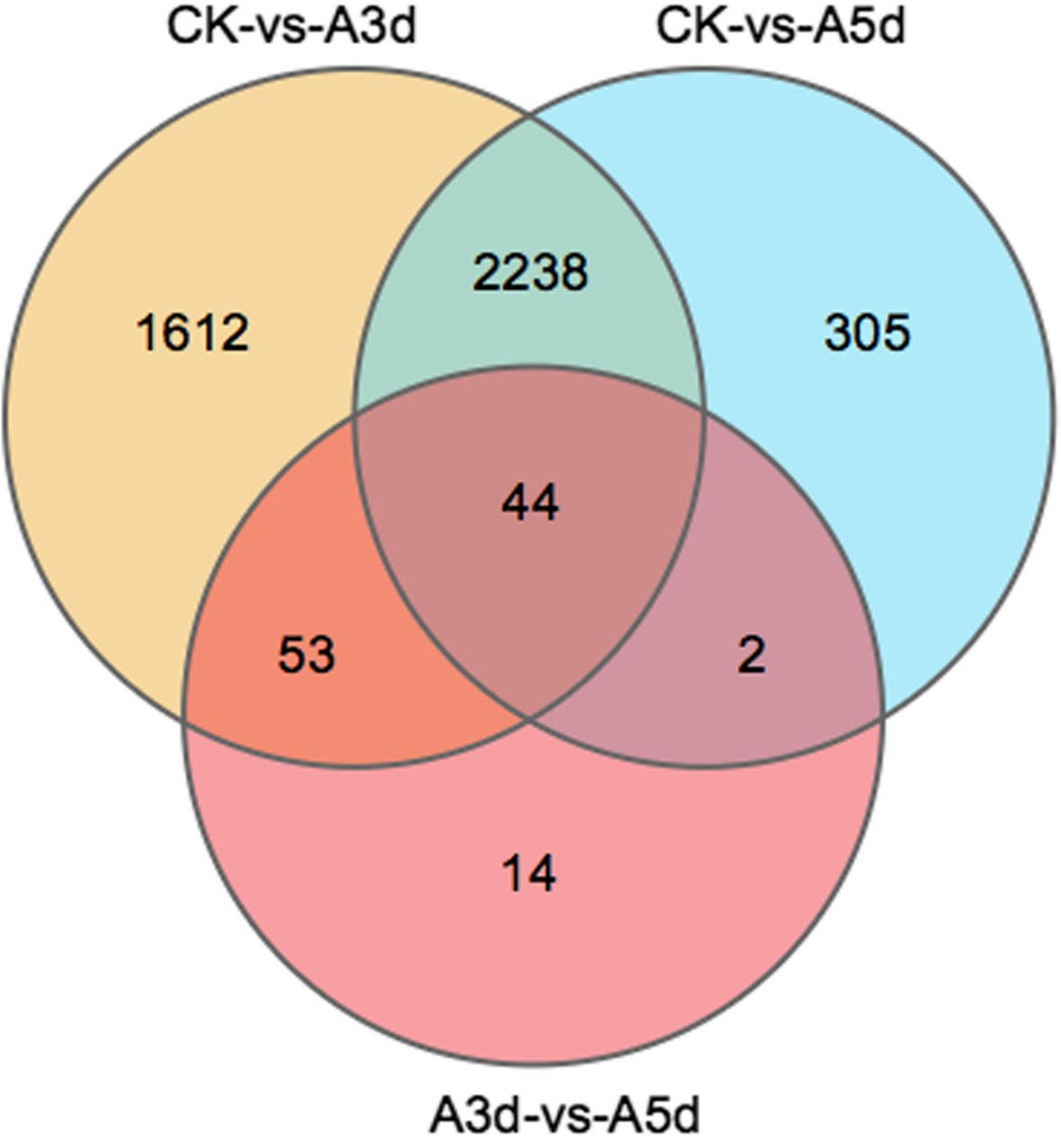
Venn map of differentially expressed genes (DEGs) between CK VS A3 d, CK VS A5d and A3d VS A5 d. (CK) non-infected rose leaves, (A3 d) *M. rosivorum* inoculated *R. longicuspis* with 3 ds, (A5 d) *M. rosivorum* inoculated *R. longicuspis with* 5 ds

To obtain a comprehensive view of the gene expression profile associated with the response of *R. longicuspis* to *M. rosivorum*, we used DESeq2 to identify the DEGs. Based on the filtering parameters of FDR < 0.05 and |log2FC| > 1, the expression levels of 2845 (1186 upregulated, 1659 downregulated), 2627 (886 upregulated, 1741 downregulated) and 466 (178 upregulated, 288 downregulated) genes were found to differ significantly in the CK-vs.-A3 d, CK-vs.-A5 d, and A3 d-vs.-A5 d groups, respectively. Among those genes, 998 and 904 DEGs were expressed in A3 d and A5 d, respectively (Fig. 2). These results indicate that a large number of genes were upregulated and that few new DEGs were expressed in the *R. longicuspis*-*M. rosivorum* interaction.

**Fig. 3:**
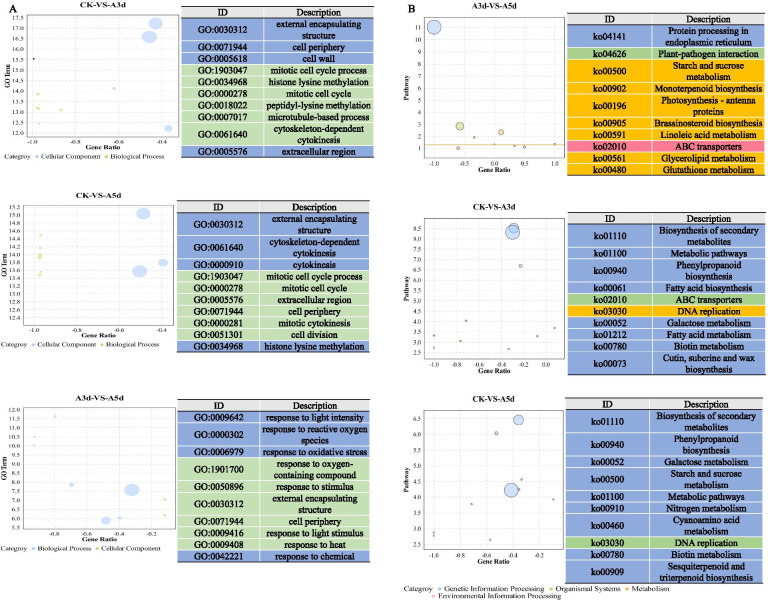
GO (**A**) and KEGG (**B**) analysis based on DEGs in CK VS A3 d, CK VS A5 d, and A3 d VS A5 d. (CK) non-infected rose leaves, (A3 d) *M. rosivorum* inoculated *R. longicuspis* with 3 ds, (A5 d) *M. rosivorum* inoculated *R. longicuspis with* 5 ds

To understand the functions of the DEGs associated with *M. rosivorum*, those DEGs were annotated using GOseq. This annotation resulted in three major categories: biological processes, cellular components, and molecular functions. A comparison of the CK at 3 d and 5 d indicated that most of the DEGs were enriched in the ‘external encapsulating structure’, ‘cell periphery’, ‘cell wall’, ‘cytoskeleton-dependent cytokinesis’ and ‘histone lysine methylation’ categories and other terms. A comparison of 3 d with 5 d showed that most of the DEGs were enriched in the ‘response to reactive oxygen species’, ‘response to oxidative stress’, ‘response to oxygen-containing compound’ and other terms (Fig. 3A). To better understand the main pathways activated under *M. rosivorum* stress, we conducted a Kyoto Encyclopaedia of Genes and Genomes (KEGG) enrichment analysis of the DEGs. Between the CK and A3 d libraries, 492 DEGs were assigned to 113 KEGG pathways. Between the CK and A5 d libraries, 484 DEGs were assigned to 109 KEGG pathways. Among these pathways, ‘biosynthesis of secondary metabolites’, ‘metabolic pathways’, ‘phenylpropanoid biosynthesis’, ‘fatty acid biosynthesis’, ‘galactose metabolism’, ‘cutin, suberine and wax biosynthesis’, ‘cyanoamino acid metabolism’ and ‘sesquiterpenoid and triterpenoid biosynthesis’ were significantly enriched. Between the A3 d and A5 d libraries, 105 DEGs were assigned to 51 KEGG pathways. Among these pathways, some were related to plant insect resistance pathways associated with the terms ‘plant-pathogen interaction’, ‘starch and sucrose metabolism’, ‘monoterpenoid biosynthesis’ and ‘brassinosteroid biosynthesis’ (Fig. 3B). Taken together, the results showed that under *M. rosivorum* stress, the antioxidant system, terpenoid synthesis and secondary metabolite biosynthesis of *R. longicuspis* were activated to reduce aphid damage in *R. longicuspis*.

**Fig. 4:**
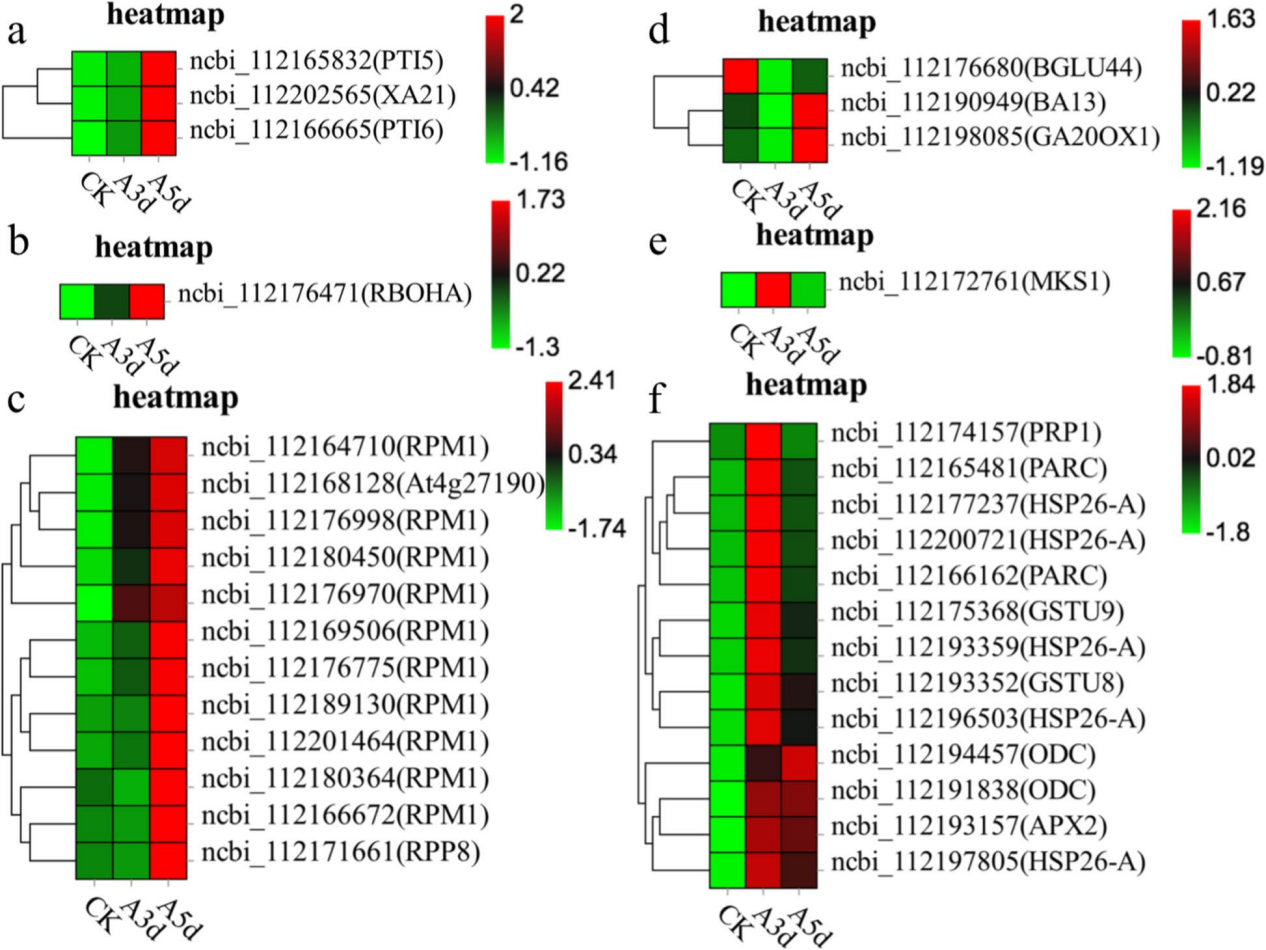
**a** Heatmap of PTI genes in *R. longicuspis* under *M. rosivorum* stress. **b** Heatmap of ROS metabolic pathway gene in *R. longicuspis* under *M. rosivorum* stress. **c** Heatmap of resistance protein genes in *R. longicuspis* under *M. rosivorum* stress. **d** Heatmap of biosynthesis of secondary metabolism genes in *R. longicuspis* under *M. rosivorum* stress. **e** Heatmap of MAPK pathway gene in *R. longicuspis* under *M. rosivorum* stress. **f** Heatmap of GST genes in *R. longicuspis* under *M. rosivorum* stress. The bar represents the scale of the expression levels for each gene (FPKM) in the different treatments, as indicated by red/green rectangles. Genes in red show upregulation, and those in green show downregulation

Heatmaps of DEG subclusters were developed to better understand the key DEGs associated with the resistance of *R. longicuspis* to *M. rosivorum.* The resulting heatmaps showed the DEGs involved in *R. longicuspis*-*M. rosivorum* interactions. Based on their functional annotation, these genes included 1 reactive oxygen species metabolic pathway gene, 3 secondary metabolite synthesis genes, and 13 glutathione metabolism genes, which are shown in the heatmap (Fig. 4). These findings indicated that *R. longicuspis* responds to *M. rosivorum* by activating the expression of signal transduction pathway genes, secondary metabolite synthesis genes, antioxidant stress genes and disease resistance genes.

### Validation of candidate DEGs based on qPCR analysis

To validate the reliability of the DEGs obtained from the RNA-Seq analyses, the expression levels of 6 candidate genes were analysed using qPCR. These genes included the 1 α-linolenic acid metabolism gene (*Rl AOC*), 1 starch and sucrose metabolism gene (*Rl BGLU11*), 2 plant-type cell wall modification genes (*Rl EXPB3* and *Rl EXPA1*), 1 hormone response gene (*Rl MBF1C*) and 1 homologous recombination gene. The correlation coefficients (r) between the RNA-Seq and qPCR results were calculated for these DEGs (Fig. S2; Table S2). The results showed that the correlation coefficients were greater than 0.75, indicating that the RNA-Seq data were reliable.

### Overview of the metabonomic analysis

To understand the changes in metabolites and the possible defence mechanisms of *R. longicuspis* to infection by *M. rosivorum*, a metabolite profiling analysis of the *R. longicuspis* leaf samples (CK, A3 d, A5 d) was performed. A total of 758 metabolites were detected in all samples, and they could be divided into 35 groups (Table S3). The principal component analysis (PCA) showed that the repeatability of different treatments was good. Orthogonal partial least squares discriminant analysis (OPLS-DA) showed that the results of OPLS-DA analysis could be used for subsequent model tests and differential metabolite analysis. Between the CK and A3 d treatments, 31 were upregulated and 34 were downregulated. Between the CK and A5 d treatments, 32 were upregulated and 38 were downregulated. The levels of α-linolenic acid*, γ-linolenic acid*, 2-O-salicyl-6-O-galloyl-D-glucose, scopoletin-7-O-glucuronide, and geniposide increased significantly with the extension of infection time (Table S4). This finding indicated that these secondary metabolites were involved in the resistance response of *R. longicuspis* to *M. rosivorum.*

To identify the main pathways that *R. longicuspis* uses to respond to *M. rosivorum,* we mapped the differentially expressed metabolites based on a KEGG biological pathway analysis. A total of 283 metabolites were assigned to 94 KEGG pathways, including ‘metabolic pathways’ (59.72%), ‘biosynthesis of secondary metabolites’ (36.75%), and ‘biosynthesis of antibiotics’ (18.73%), among others (Table S5). Sixty-eight significantly differentially expressed metabolites between the CK and A3 d treatments were assigned to 43 KEGG pathways, including ‘alpha-linolenic acid metabolism’, ‘cyanoamino acid metabolism’, ‘tropane, piperidine and pyridine alkaloid biosynthesis’ and others (Table S6). Seventy significantly differentially expressed metabolites between the CK and A5 d treatments were assigned to 43 KEGG pathways, including ‘aminoacyl-tRNA biosynthesis’, ‘cyanoamino acid metabolism’, ‘glucosinolate biosynthesis’ and others (Table S7). Twenty-six significantly differentially expressed metabolites between the A3 d and A5 d treatments were assigned to 15 KEGG pathways, including ‘alpha-linolenic acid metabolism’ and ‘biosynthesis of unsaturated fatty acids’ (Table S8). The results showed that the metabolic pathways related to disease resistance were significantly enriched, indicating that the defence mechanism of *R. longicuspis* was activated under *M. rosivorum* stress.

### Glucosinolate biosynthesis

**Fig. 5:**
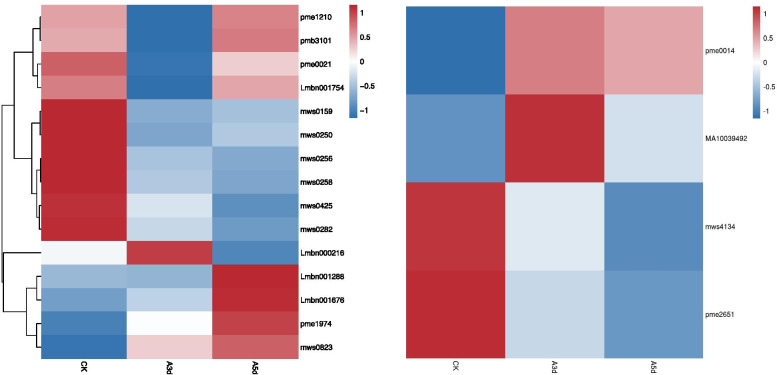
Heatmap of metabolites in glucosinolate metabolism (left) and glutathione metabolism (right) pathway. The abscissa represents the sample name and hierarchical metabolite code

The glucosinolate biosynthesis pathway is involved in plant defence against insects. In our research, 15 metabolites of the glucosinolate biosynthesis pathway exhibited different levels in different treatments (Fig. 5). Among those metabolites, the content levels of L-isoleucine*, L-valine and L-tyrosine were decreased significantly; moreover, compared with the CK, the levels in A3 d and A5 d decreased by factors of 1.62, 2.11, and 1.64 and 2.63, 3.06, and 1.64, respectively. In addition, the contents of pyruvic acid, 3-methyl-2-oxobutanoic acid*, (S)-2-hydroxy-2-methyl-3-oxobutanoic acid*, (R)-3-hydroxy-3-methyl-2-oxopentanoic acid* and 2-isopropylmalic acid increased. These results indicate that the glucosinolate biosynthesis pathway may be positively involved in the interaction between *R. longicuspis* and *M. rosivorum.*

### Glutathione metabolism

Glutathione plays an important role in plant resistance to external stress and reactive oxygen species injury. The ratio of reduced glutathione to oxidized glutathione is one of the important indicators of glutathione activity. In our research, the contents of oxiglutatione and NADP (nicotinamide adenine dinucleotide phosphate) decreased continuously (Fig. 5). Compared with the CK, oxiglutatione decreased by 35.15 and 55.29% in A3 d and A5 d, respectively, and NADP decreased by 12.92 and 17.63% in A3 d and A5 d, respectively. The content level of dehydroascorbic acid first increased and then decreased. Compared with the CK, dehydroascorbic acid increased by 12.42 and 3.86% in A3 d and A5 d, respectively, and L-glutamic acid* increased by 12.08 and 10.84% in A3 d and A5 d, respectively. The results showed that *M. rosivorum* infection induced the antioxidant system of *R. longicuspis* and dehydroascorbic acid and GSH were involved in *R. longicuspis* resistance to oxidative stress caused by *M. rosivorum..*

### Conjoint analysis

**Fig. 6:**
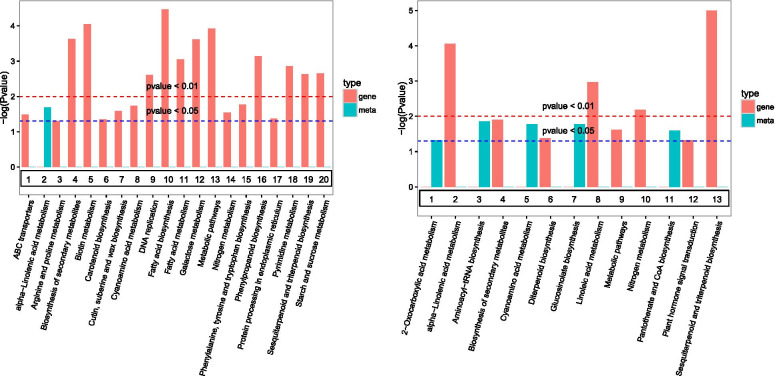
Joint KEGG enrichment *p*-value histogram in CK-VS-A3 d (left) and CK-VS-A5 d (right)

A conjoint KEGG enrichment analysis showed 84 comapped pathways, with 35 and 16 comapping pathways between CK-vs.-A3 d and CK-vs.-A5 d and their metabolites, respectively. Interestingly, of these co-mapped pathways, ‘fatty acid biosynthesis’, ‘metabolic pathways’ and ‘biosynthesis of secondary metabolites’ were significantly enriched in CK-vs.-A3 d, and ‘2-oxocarboxylic acid metabolism’, ‘alpha-linolenic acid metabolism’, ‘sesquiterpenoid and triterpenoid biosynthesis’, ‘linolenic acid metabolism’ and ‘glucosinolate biosynthesis’ were significantly enriched in CK-vs.-A5 d (Fig. 6), indicating that the metabolic pathway related to aphid resistance was activated during aphid stress.

Based on the the two-way orthogonal partial least squares (O2PLS) model, the combined analysis of transcriptomics and metabonomic data showed that the model was reliable (R2 > 0.84). The Pearson correlation coefficients showed that the differential expression patterns of DEGs and metabolites were consistent. The correlations between the top 250 DEGs and their metabolites were further selected and are represented as a heat map (Fig. S3).

## Discussion

### RNA-seq study for disease resistance

Aphids damage the new shoots, young leaves and flower buds of rose plants, which can lead to the decline of rose growth, hinder normal growth or blooming, and seriously affect the ornamental value [5]. Insect herbivory induces several internal signals from wounded plant tissues, including calcium ion fluxes, phosphorylation cascades and systemic and jasmonate signalling. Moreover, plants also induce defence compounds to strengthen their defence [24]. Tan et al. [25] found that rice infected with *Nilaparvata lugens* induced metabolic processes, cellular development, cell wall organization, cellular component movement and hormone transport, and certain primary and secondary metabolite synthesis processes. Li et al. [26] found that *Acyrthosiphon pisum* infection induced the activity of defence enzymes in *Medicago sativa* and activated metabolic pathways such as ‘phenylpropane biosynthesis’ and ‘phenylalanine metabolism’. Serba et al. [27] found that after grain sorghum was infected by *Melanaphis sacchari*, its gene expression of regulatory protein and lipid synthesis, cell catabolism, cell communication, transcription initiation and autophagy was upregulated, which was involved in the plant’s defence against aphids. Under *M. rosivorum* stress, the expression of a series of antioxidant-, plant hormone- and disease resistance-related genes is induced in *R. longicuspis*, including ROS production genes, GST genes, PTI genes and RPM genes, which were significantly upregulated (Fig. 4). Moreover, DEGs were significantly enriched in ‘fatty acid biosynthesis’, ‘biosynthesis of secondary metabolites’, ‘phenylpropanoid biosynthesis’, ‘starch and sucrose metabolism’, ‘sesquiterpenoid and triterpenoid biosynthesis’, ‘phenylalanine, tyrosine and tryptophan biosynthesis’, ‘cutin, suberine and wax biosynthesis’, ‘alpha-linolenic acid metabolism’ and other pathways related to plant disease and insect resistance. Interestingly, ‘phosphatidylinositol signalling system’ and ‘MAPK signalling pathway - plant’ pathway was also activated. All of these transcriptomic data indicated that multiple processes in *R. longicuspis* are associated with plant defence against aphids, which is consistent with the fact that the plants have evolved a complex defence mechanism [28, 29]. This result revealed that *M. rosivorum* infection induced a change in reactive oxygen species and led to effector-triggered immunity in *R. longicuspis*.

Brassinosteroids (BRs) play an important role in plant growth, developmental processes, and responses to pathogen infection, although the role of BRs in the interactions between plants and insects is still unclear. Liu et al. [30] found that brassinosteroids (BRs) could enhance *Rhagoletis batava obseuriosa* resistance in sea buckthorn (*Hippophae rhamnoides*). Liao et al. [31] found that BR contributes to the growth-defence tradeoff by suppressing the expression of defensin and glucosinolate biosynthesis genes. Pan et al. [32] demonstrated that BRs promote the susceptibility of rice (*Oryza sativa*) plants to BPH (*Nilaparvata lugens*) by modulating the SA and JA pathways. Interestingly, the expression of genes in the ‘brassinosteroid biosynthesis’ pathway increased significantly in A3 d vs. A5 d. Therefore, the mechanism of brassinolate on the interaction between rose and aphids is worthy of further study.

As important regulatory factors, transcription factors play an important role in regulating the plant response to environmental signal stimulation. Related transcription factors can be roughly divided into six categories: AP2/ERF family, bHLH family, bZIP family, MYB family, NAC family and WRKY family [33, 34]. Overexpression of *CmWRKY48* in chrysanthemum can improve its defence against aphids [35]. *Arabidopsis MYB102* increases host susceptibility to GPA through ET-dependent signalling pathways [36]. Jacques et al. [37] found that NAC, AP2 domain and ERF transcription factors are important regulators of *Medicago truncatula’s* defence against aphids. In our study, the expression of 5 transcription factors, namely, 1 bZIP transcription factor (*Rl ABF2*) and 4 MYB transcription factors (*Rl RVE7/8, Rl PHL5 and Rl MYR2*), was significantly higher than that in the control (Fig. S4). These results suggest that these five transcription factors may be involved in the response of rose to aphids. Taken together, the results show that under *M. rosivorum* stress, the *R. longicuspis* signal transduction pathway is activated to induce related transcription factors to regulate the expression of downstream disease resistance genes.

### Metabolomics study for disease resistance

Secondary metabolites, such as flavonoids, terpenoids, phenols and alkaloids, have the characteristics of antibiotics and thus play an important role in the defence of phytophagous insects [38, 39]. Terpenoids, flavonoids and tannins enhance plant defence against aphids by affecting aphid colonization [19, 40–42]. Our metabolomics results highlighted differential changes in 15 major classes of metabolites. The contents of eight secondary metabolites, including organic acids, lipids, phenolic acids, lignans and coumarins, terpenoids, tannins, flavonoids, and cis-p-coumaric acid 4-O-glucoside* increased significantly. Starch sucrose metabolism plays an important role in the defence of phytophagous insects. Previous studies have shown that the accumulation of starch in *Solanum lycopersicum* enhances its defence against peach aphids (*Myzus persicae*) [43]. In our research, the starch synthase SS1 (*Rl SS1*), trehalose 6-phosphate phosphatase (*Rl TPPA*) and β-glucosidase (*Rl BGLU11*) genes were upregulated while the contents of D-glucose 6-phosphate* and glucose-1-phosphate* genes were significantly downregulated, indicating that under aphid stress, rose enhanced its defence against aphids by promoting starch synthesis.

### Aphid stress triggered Glucosinolate accumulation

Glucosinolates can be used as biological insecticides to effectively control aphids [44]. Lei et al. [45] showed that overexpression of the *AtCCA1* gene enhanced resistance to peach aphids by increasing the content of glucosinolates in Arabidopsis (*Arabidopsis thaliana*). Kim et al. [46] found that aphids feeding on *Arabidopsis thaliana* induced the synthesis of 4-methoxyindole-3-methylthiogluconate, and exogenous 4-methoxyindole-3-methylthiogluconate could enhance its resistance to aphids. Moreover, a defensive role for indole glucosinolates is suggested by the observation that atr1D mutant plants, which overproduce indole glucosinolates, are more resistant to *M. persicae*, whereas cyp79B2 cyp79B3 double mutants, which lack indole glucosinolates, succumb to *Myzus persicae* more rapidly. Artificial diet experiments show that the reaction of indole-3-carbinol, a breakdown product of indol-3-ylmethylglucosinolate, with ascorbate, glutathione and cysteine produces diindolylmethylcysteines and other conjugates that have antifeedant effects on *M. persicae* [47]. These studies showed that glucosinolates are important chemically active substances for plant defence against aphids. Under aphid stress, the expression of N-hydroxythioamide S-beta-glucosyltransferase genes was induced, and the content levels of L-2-amino-4-methylthiobutyric acid, (S)-alpha-amino-beta-(3-indolyl)-propionic acid and tyrosine, among others, increased. Thus, the glucosinolate metabolic pathway may be involved in the resistance of *R. longicuspis* to *M. rosivorum.* Therefore, the mechanism of glucosinolates on the interaction between rose bushes and aphids is worthy of further study.

### Aphid stress triggers changes in glutathione metabolism

Glutathione plays a crucial role in the defence responses of plants to biotic stress factors [48, 49]. *Rhopalosiphum padi* and *Sitobion avenae* were shown to induce the expression of AsA-GSH cycle-related genes in maize (*Zea mays* L.) and affect the contents of reduced and oxidized ascorbic acid and glutathione [50]. Glutathione metabolic pathway genes were significantly induced and expressed in *Sorghum bicolor* under *M. sacchari* stress [51]. Pant et al. [52] found that sorghum enhances the scavenging capacity of reactive oxygen species by inducing the upregulation of glutathione metabolic pathway genes and participates in the resistance response to *M. sacchari*.. Our study showed that under aphid stress, the contents of dehydroascorbic acid and L-glutamic acid* were higher than those of the control. However, the contents of oxiglutatione and NADP were lower than that of the control. The expression of glutathione metabolic pathway genes (*Rl ODCs*, *Rl APXs*, *Rl GSTs*, and *Rl HSP26-As*, Fig. 4) was induced. *R. longicuspis* appeared to compensate for the effects of oxidative stress induced by *M. rosivorum* by the elevated expression of genes and affect the content of metabolites involved in the AsA-GSH cycle.

## Conclusion

We combined transcriptome and metabolome analyses to explore the response mechanism of *R. longicuspis* to *M. rosivorum.* Under *M. rosivorum* stress, *M. rosivorum* infection induced a series of signal transduction, MAPK signalling, inositol phosphate signalling and endogenous hormone signalling pathways and induced the expression of *RlMYBs*, *RlERFs* transcription factors and resistance related genes. Interestingly, the ‘brassinosteroid biosynthesis’ pathway was significantly enriched in A3 d-vs.-A5 d. Moreover, the defence responses included the transformation of starch and sucrose, the synthesis of terpenoids, tannins and phenolic acids, among others. According to the metabolomic data analysis, glucosinolate metabolism and the glutathione metabolic pathway were significantly enriched. Our study provides candidate genes and metabolites for rose defence against aphids. This study provides a theoretical basis for further exploring the mechanism underlying the molecular regulation of rose aphid resistance and aphid resistance breeding in the future.

## Material and methods

### Plant growth and plant infection

The plant material was identified by Professor Hongying Jian, Flower Research Institute, Yunnan Agriculture Academic Science. Based on the characteristics of its stem, leaf and flower, among others, Dr. Hongying Jian identified it as *Rosa longicuspis* according to the specimen of Rosa longifolia (voucher specimen Code: 565971) stored in the herbarium of South China Botanical Garden, Chinese Academy of Sciences. And the germplasm (No. KM-R-RL-2010) is deposited in the Germplasm Resources Nursery of Flower Research Institute, Yunnan Agriculture Academic Science, Kunming, China. Coordinates: 25°7′ 35“ N, 102°45’ 23” E. We declare that the research programme complies with relevant institutional, national and international guidelines and legislation, and we have permission to collect *R. longicuspis*.

*M. rosivorum* was applied with a brush to the stem end or undeveloped young leaves of the identified plants, and then the plants were covered with a cylinder, and the plant and aphid grew and reproduced normally [53]. Infected and control plants were individually sampled in a randomized manner from each of the three trays at 3 d and 5 d with three biological repeats for both infected and control treatments at each time point. Leaves were immediately frozen in liquid nitrogen at the time of harvesting and stored at − 80 °C.

### Observations of the number of *M. rosivorum*

The number of *M. rosivorum* individuals after inoculation for 1 d, 3 d, 5 d and 7 d were counted.

### RNA extraction, library construction and sequencing

Total RNA was extracted using a TRIzol reagent kit (Invitrogen, Carlsbad, CA, USA) according to the manufacturer’s protocol. RNA quality was assessed using an Agilent 2100 Bioanalyzer (Agilent Technologies, Palo Alto, CA, USA) and checked using RNase-free agarose gel electrophoresis. After total RNA was extracted, eukaryotic mRNA was enriched by oligo(dT) beads while prokaryotic mRNA was enriched by removing rRNA with a Ribo-Zero™ Magnetic Kit (Epicentre, Madison, WI, USA). Then, the enriched mRNA was fragmented into short fragments using fragmentation buffer and reverse transcribed into cDNA with random primers. Second-strand cDNA was synthesized by DNA polymerase I, RNase H, dNTPs and buffer. Then, the cDNA fragments were purified using a QIAquick PCR extraction kit (Qiagen, Venlo, The Netherlands), end repaired was performed, poly(A) was added, and then the fragments were ligated to Illumina sequencing adapters. The ligation products were size-selected by agarose gel electrophoresis, PCR-amplified, and sequenced using an Illumina HiSeq2500 [54].

### Transcriptomic data analysis

To obtain high-quality clean reads, we removed the adaptor-containing sequences, poly-N, and low-quality reads. The remaining clean reads were further used in the assembly and gene abundance calculation. Then, clean reads were mapped to the reference genome using the HISAT2 tool [55]. For each transcription region, an FPKM (fragment per kilobase of transcript per million mapped reads) value was calculated to quantify its expression abundance and variations using StringTie software [56, 57].

Differential expression analyses among the three treatments (CK-vs.-A3 d, CK-vs.-A5 d, and A3 d-vs.-A5 d with three biological replicates per treatment) were conducted using DESeq2 software [58]. Genes/transcripts with a false discovery rate (FDR) below 0.05 and absolute fold change of ≥2 were considered differentially expressed genes/transcripts.

A GO enrichment analysis identified all terms that were significantly enriched in the DEGs compared to the genome background and filtered the DEGs that corresponded to biological functions. KEGG [59–61] is the major public pathway-related database, and a KEGG pathway enrichment analysis identified significantly enriched metabolic pathways or signal transduction pathways in the DEGs compared with the whole genome background.

### Quantitative real-time PCR validation

qPCR was used to validate the RNA-seq data for 6 different genes. Specific primers were designed using Premier 5 software (Premier Biosoft, Palo Alto, CA, USA). The RNA samples were used to synthesize cDNA, and a Step OnePlus Real-Time Fluorescent Quantitative PCR system (Trans Start® Green qPCR Super Mix) was used to monitor the amount of DNA. Assays of each gene were repeated three times. Quantification was evaluated using the 2^−(ΔΔCt)^ method.

### Extraction and quantification of metabolites

Metabolites were extracted from leaves with three replicates per treatment. The extracted were analysed using an LC-ESI-MS/MS system (UPLC, Shim-pack UFLC SHIMADZU CBM30A, http://www.shimadzu.com.cn/; MS/MS (Applied Biosystems 6500 QTRAP)). LIT and triple quadrupole (QQQ) scans were acquired on a triple quadrupole-linear ion trap mass spectrometer (Q TRAP) [62] and an AB Sciex QTRAP6500 System, equipped with an ESI-Turbo Ion-Spray interface, operating in positive ion mode and controlled by Analyst 1.6.1 software (AB Sciex). The operation parameters were as follows: ESI source temperature 500 °C; ion spray voltage (IS) 5500 V; curtain gas (CUR) 25 psi; and collision-activated dissociation (CAD). QQQ scans were acquired as MRM experiments with optimized decluttering potential (DP) and collision energy (CE) for each individual MRM transition [63]. The m/z range was set between 50 and 1000.

Metabolites were identified by searching internal databases and public databases (Mass Bank, KN Ap Sac K, HMDB, Mo to DB, and METLIN) and comparing the m/z values, RT values, and fragmentation patterns with the standards [64].

### Metabolomic data analysis

A T-test was performed, and metabolites with a *P* value of < 0.05 and variable important in projection (VIP) ≥ 1 were considered differential metabolites between the groups. We constructed metabolic pathways based on the information in the KEGG database.

### Combined metabolomic and transcriptomic analysis

To reveal the regulatory and influencing mechanism between gene expression and metabolite production, we analysed three models based on gene expression and metabolite abundance. The correlation between the top 250 differentially expressed genes and their metabolites was used to draw a heatmap.

## Supplementary Information

**Additional file 1: Figure S1.** Sample correlation heat map.

**Additional file 2: Figure S2.** The correlations between the expression profiles of the 6 DEGs were determined by RNA-Seq and qPCR analysis.

**Additional file 3: Figure S3.** Heatmap of the top 250 DEGs and their metabolites.

**Additional file 4: Figure S4.** Heatmap of 5 transcription factors.

**Additional file 5: Table S1.** Unigene annotation chart.

**Additional file 6: Table S2.** qPCR primer.

**Additional file 7: Table S3.** List of all detected metabolites.

**Additional file 8: Table S4.** DEMs of *M. rosivorum* in response to *R. longicuspis.*

**Additional file 9: Table S5.** KEGG annotation of metabolites.

**Additional file 10: Table S6.** KEGG annotation of differentially expressed metabolites from CK-vs.-A3 d.

**Additional file 11: Table S7.** KEGG annotation of differentially expressed metabolites from CK-vs.-A5 d.

**Additional file 12: Table S8.** KEGG annotation of differentially expressed metabolites from A3 d-vs.-A5 d.

## Data Availability

The Sequence dataset used and/or analyzed during the current study are available from the corresponding author on reasonable request.
