# Supplementary figures and images for: Transcriptomic and metabolomic changes triggered by Macrosiphum rosivorum in rose (Rosa longicuspis)
Source: BMC Genomics. 2021 Dec 9;22:885. doi: 10.1186/s12864-021-08198-6 (PMC8656021; doi:10.1186/s12864-021-08198-6)

Sample correlation heat map

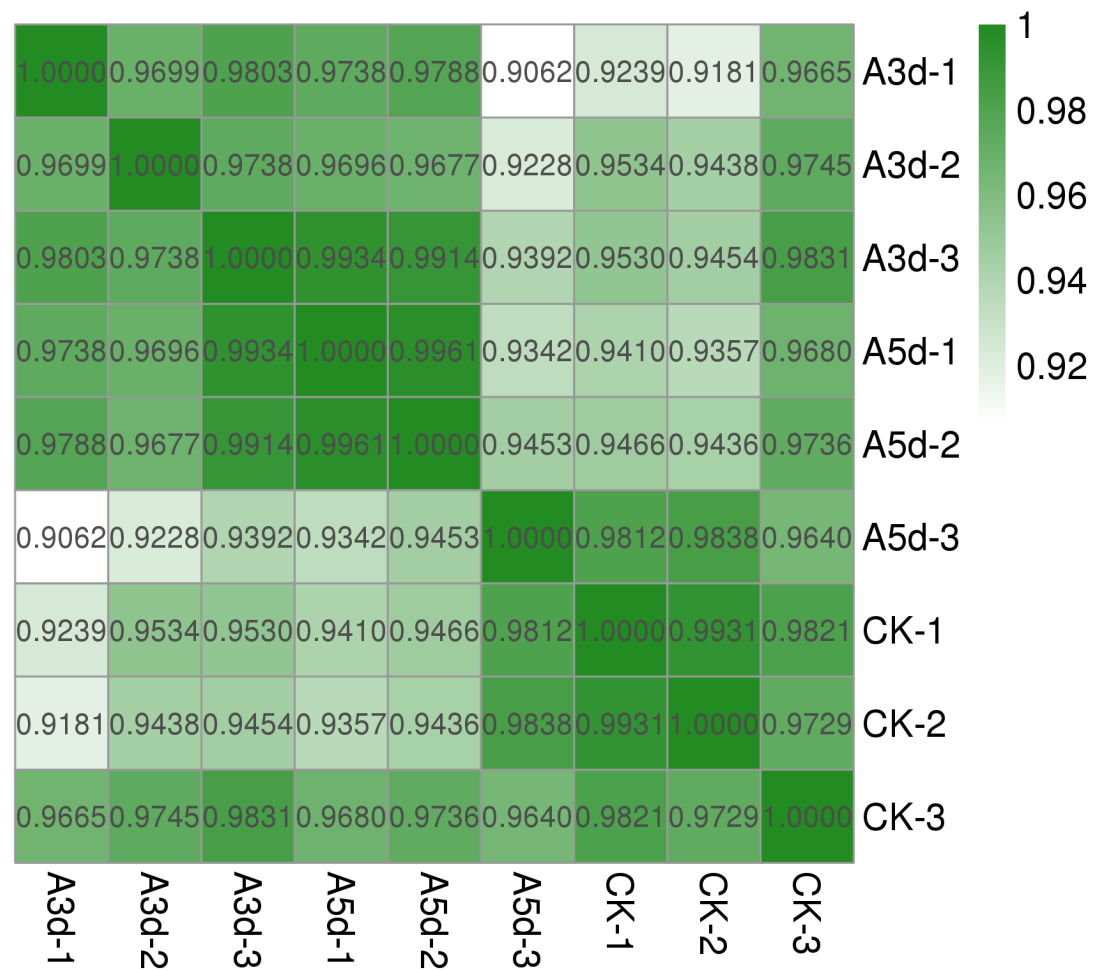

Supplement: Supplementary file 1 — Additional file 1: Figure S1. Sample correlation heat map. [file 12864_2021_8198_MOESM1_ESM.pdf]

Heatmap of the top 250 DEGs and their metabolites

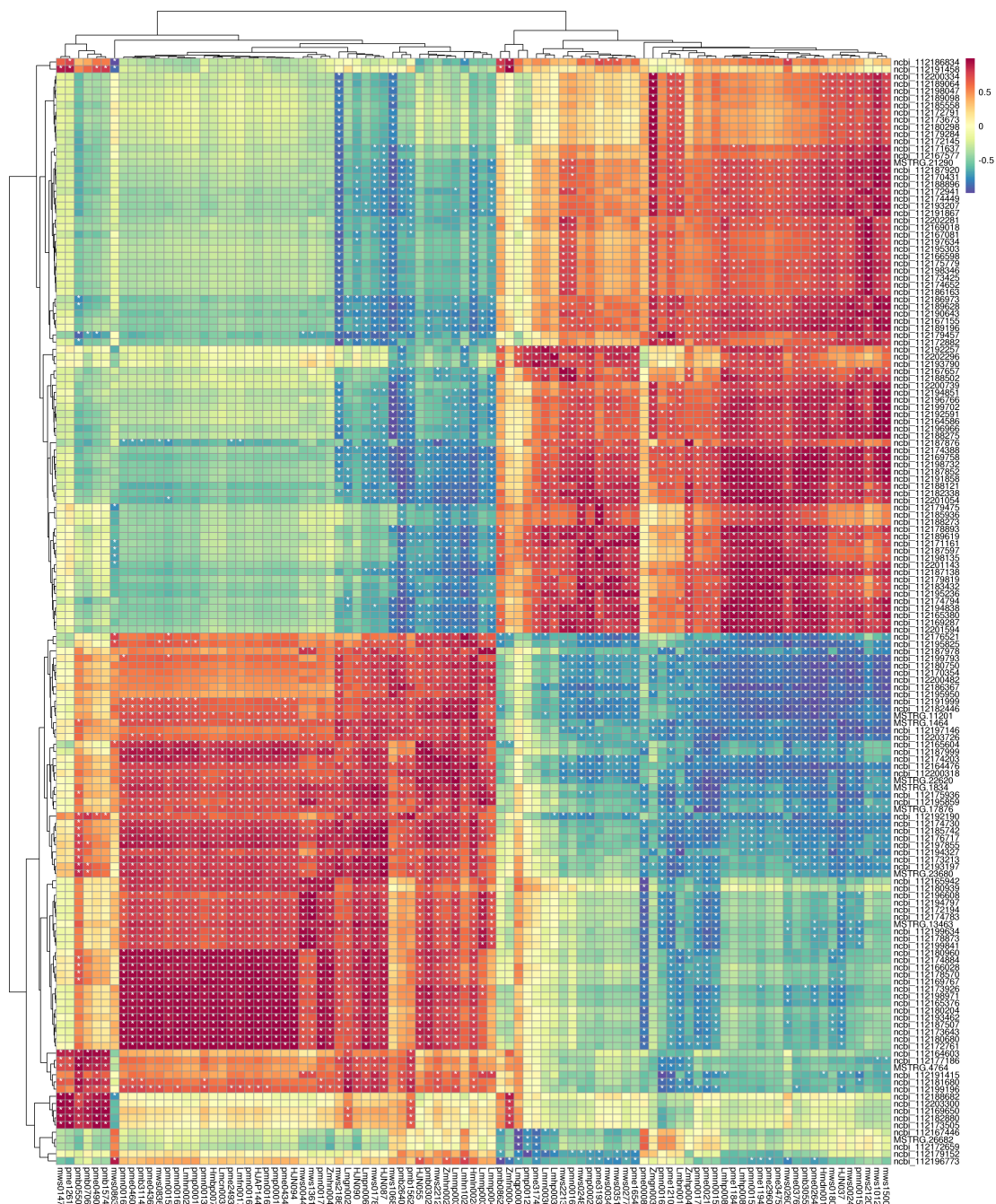

Supplement: Supplementary file 3 — Additional file 3: Figure S3. Heatmap of the top 250 DEGs and their metabolites. [file 12864_2021_8198_MOESM3_ESM.pdf]

Heatmap of 5 transcription factors

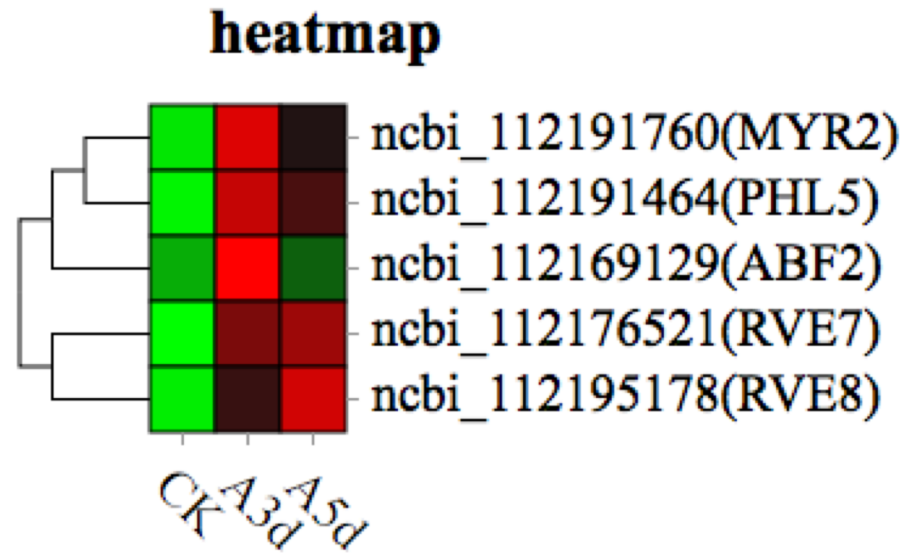

Supplement: Supplementary file 4 — Additional file 4: Figure S4. Heatmap of 5 transcription factors. [file 12864_2021_8198_MOESM4_ESM.pdf]
