# Supplementary material for: Transcriptomic and metabolomic changes triggered by Macrosiphum rosivorum in rose (Rosa longicuspis)
Source: BMC Genomics. 2021 Dec 9;22:885. doi: 10.1186/s12864-021-08198-6 (PMC8656021; doi:10.1186/s12864-021-08198-6)

The correlations between the expression profiles of the 6 DEGs were determined by RNA-Seq and qPCR analysis

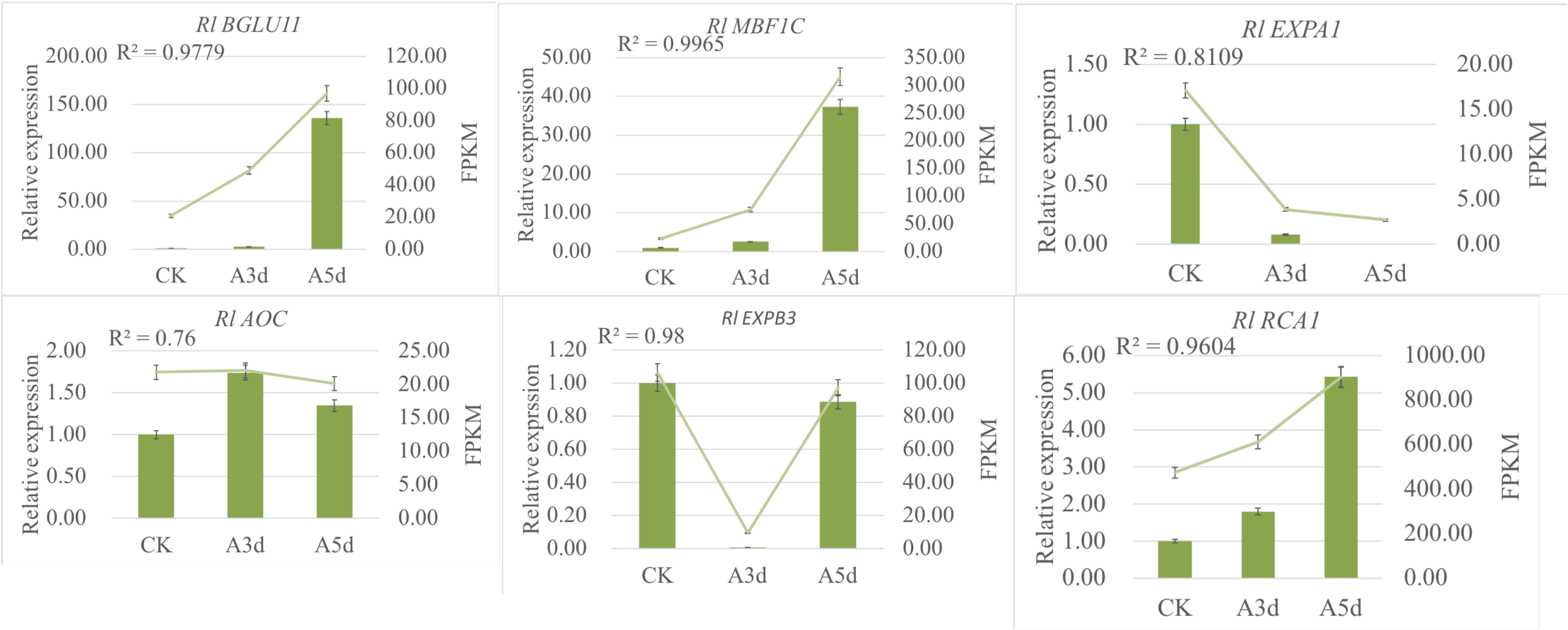

Supplement: Supplementary file 2 — Additional file 2: Figure S2. The correlations between the expression profiles of the 6 DEGs were determined by RNA-Seq and qPCR analysis. [file 12864_2021_8198_MOESM2_ESM.pdf]
